# Localized Hidradenocarcinoma of the Scalp: A Case Report

**DOI:** 10.7759/cureus.38675

**Published:** 2023-05-07

**Authors:** Sofia Elouaouch, Zahira El Youssi, Hanane Mansouri, Miry Nadir, Amal Bennani, Mohammed Amine Guerrouaz, Mohamed Moukhlissi, Soufiane Berhili, Loubna Mezouar

**Affiliations:** 1 Radiation Oncology, Mohammed VI University Hospital, Faculty of Medicine and Pharmacy of Oujda, Mohammed First University of Oujda, Oujda, MAR; 2 Anatomopathology, Faculty of Medicine and Pharmacy of Oujda, Mohammed First University of Oujda, Oujda, MAR; 3 Radiation Therapy, Mohammed VI University Hospital, Oujda, MAR

**Keywords:** localized form, surgery, radiotherapy, scalp, hidradenocarcinoma

## Abstract

Hidradenocarcinomas or malignant hidradenomas are tumors developed from the sweat glands, in particular, the eccrine glands. It is a rare entity of skin tumors and frequently appears de novo with a slight female predominance and an average age of 50 years at diagnosis. We report the case of a 57-year-old woman treated for localized hidradenocarcinoma of the scalp, successfully managed by surgery and adjuvant radiotherapy.

## Introduction

Hidradenocarcinoma is a rare malignant entity first described in 1954 by Keasbey and Hadley. It is an adnexal tumor that typically develops de novo at the expense of eccrine sweat glands and corresponds to only 6% of all malignant eccrine tumors. Clinically, it presents as a solitary, asymptomatic, slow-growing skin lesion with an elective site in the head and neck or extremities. The tumor is characterized by the frequency of locoregional recurrences and distant metastases [1,2]. The prognosis remains poor, with a five-year survival rate of 30% and high rates of distant metastases and recurrence [3].

## Case presentation

**Figure 1:**
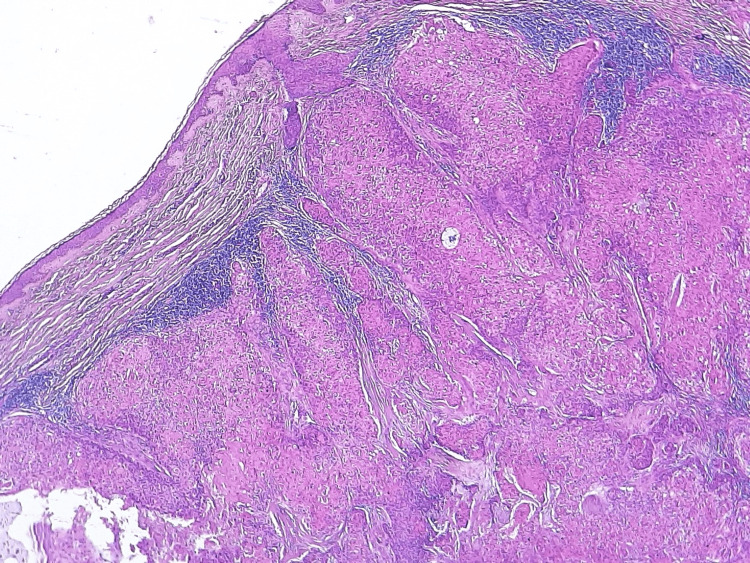
Pathology findings (H&E, x40) Photomicrograph showing poorly circumscribed dermal proliferation arranged in lobules and trabeculae and containing multiple cystic spaces.

**Figure 2:**
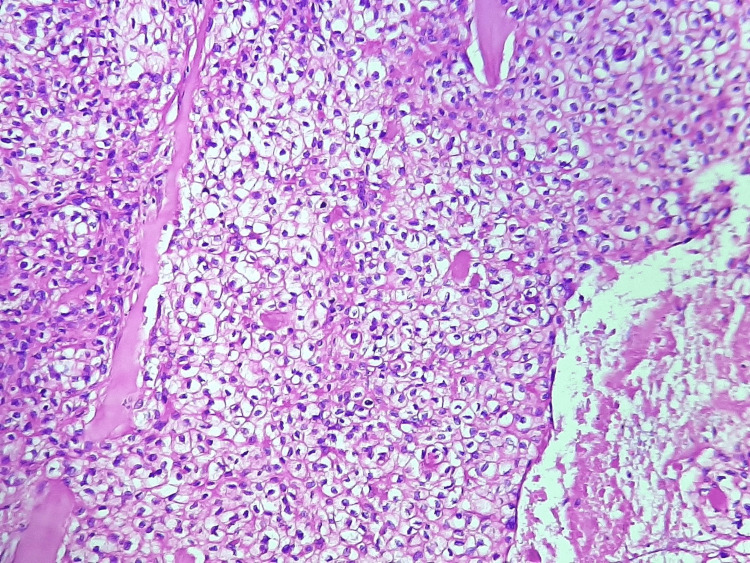
Pathology findings (H&E, x200) Tumor cells are atypical, with abundant clear to eosinophilic cytoplasm and irregular nuclei. Numerous mitotic figures are seen. Tumor necrosis is also present.

Our patient was a 57-year-old female with no significant pathological history, presenting for 10 years, a single lesion in the form of a firm and painless patch of the scalp resected at that time but without any documentation. The evolution of the symptomatology was marked by the reappearance of the lesion whose biopsy returned without signs of malignancy. The current history was characterized by a gradual increase in the size of the scalp lesion. The patient was in good general condition (WHO=0) and the somatic examination was completely normal. The cervical-thoracic-abdominal-pelvic scan was negative, with no evidence of metastasis. The patient underwent surgical resection (R1) with pathological examination aimed at a whitish subcutaneous lesion, measuring 0.7 cm in contact with the left limit, 1 mm from the deep limit, 8 mm from the anterior limit, 7 mm from the posterior limit, and 2 mm from the right limit identified histologically as hidradenocarcinoma. Other pathological features such as lymphovascular space invasion, perineural invasion, depth of invasion, and anaplasic character were negative (Figures 1, 2). Finally, the patient underwent normal-fractionated three-dimensional conformal radiotherapy as an adjuvant treatment, at a total dose of 66 Gy in 33 fractions of 2 Gy. One year later, the patient is in good control of her disease.

## Discussion

Malignant hidradenocarcinoma is a rare and very aggressive primary skin tumor. Its prognosis remains poor due to high rates of recurrence and metastasis. It appears to be slightly more common in adult females than in males, with an average age of 50 years, but cases were also reported in children and neonates. While it is a tumor that classically develops de novo, hidradenocarcinoma can be secondary to a malignant transformation of the hidradenoma, which is a benign tumor, from which comes its second name of malignant hidradenoma [4]. Clinically, hidradenoma and hidradenocarcinoma take on atypical appearances. These lesions often appear as a single painless and fixed firm nodule or plaque [5]. However, they can be multilobular, ulcerated, or fissured with cystic spaces [6]. Common differential diagnosis includes cutaneous tuberculosis, protuberant dermatofibrosarcoma, infundibular cysts, pilar cysts, pyogenic granulomas, dermatofibroma, and glomus tumor. Hidradenocarcinoma can also be clinically confused with malignancies such as basal cell carcinoma, squamous cell carcinoma, and melanoma [7].

The diagnosis is based on the presence of histological criteria such as lymphovascular invasion in the surrounding tissues, mitosis of clear cells, high mitotic activity, and loss of circumscription. Immunohistochemical found variability in the expression of hormone receptors, EGFR and HER-2, PIK3CA, AKT-1, and TP53 mutations were also detected in some cases [8,9]. The treatment of choice is complete surgical excision of the lesion with wide margins of safety associated with dissection of the regional lymph nodes followed by adjuvant radiotherapy [9]. However, the exact extent of safe surgical margins remains unspecified in the literature. Adjuvant radiotherapy is indicated in the treatment of non-metastatic patients with adverse risk factors for local recurrence, which are: positive resection margins, presence of vascular emboli, invasion or perineural engrainment, depth of infiltration, dermal lymphatic invasion, and highly anaplastic morphology. In addition, radiotherapy has been shown to be necessary when surgery is impossible, either because the tumor is not resectable or when revision surgery (theoretically mandatory after incomplete primary surgery) is impossible or entails important local complications.

The use of adjuvant chemotherapy has not been shown to be effective [10]. The prognosis of patients with hidradenocarcinoma remains poor, with a five-year survival rate after surgery of less than 30% [11], and a local recurrence rate ranging between 10% and 50% after surgery [12].

## Conclusions

Hidradenocarcinoma has long been considered an aggressive skin tumor due to its propensity for recurrence. Due to its rarity, there is no standard for treatment. Radiotherapy as an adjuvant treatment to primary surgery is necessary for the presence of pathological features on the anatomopathological examination at the localized stage for better local control. However, prospective studies with a large number of cases are needed to standardize patient management with these rare tumors.
